# Association between long working hours and mental health among nurses in China under COVID-19 pandemic: based on a large cross-sectional study

**DOI:** 10.1186/s12888-023-04722-y

**Published:** 2023-04-07

**Authors:** Hongwei Che, Huiying Wu, Yu Qiao, Bonan Luan, Qingyun Zhao, Hongyan Wang

**Affiliations:** 1 Department of Operating Room, Shengjing Hospital of China Medical University, Shenyang, Liaoning P. R. China; 2 Department of Urology, Shengjing Hospital of China Medical University, Shenyang, Liaoning P. R. China; 3 Department of Thoracic Surgery, Shengjing Hospital of China Medical University, 36 Sanhao Street, 110004 Shenyang, Liaoning P. R. China

**Keywords:** Working hours, Depression, Anxiety, Nurses, COVID-19

## Abstract

**Objective:**

Nurses were more likely to experience mental disorders due to long working hours and irregular schedules. However, studies addressing this issue are scarce; therefore, we aimed to investigate the association between long working hours and mental health in Chinese nurses during the coronavirus disease pandemic.

**Methods:**

A cross-sectional study was conducted with 2,811 nurses at a tertiary hospital in China from March to April 2022. We collected data on demographic, psychological characteristics, dietary habits, life, and work-related factors using a self-reported questionnaire and measured mental health using Patient Health Questionnaire-9 and General Anxiety Disorder-7. Binary logistic regression to determine adjusted odds ratios and 95% confidence intervals.

**Results:**

The effective response rates were 81.48%, 7.80% (219), and 6.70% (189) of the respondents who reported depression and anxiety, respectively. We categorized the weekly working hours by quartiles. Compared with the lowest quartile, the odds ratios and 95% confidence intervals across the quartiles for depression after adjustment were 0.98 (0.69, 1.40), 10.58 (2.78, 40.32), and 1.79 (0.81, 3.97) respectively, the *P* for trend was 0.002. The odds ratios across the quartiles for anxiety after adjustment were 0.87 (0.59, 1.30), 8.69 (2.13, 35.46), and 2.67 (1.26, 5.62), respectively, and the *P* for trend was 0.008.

**Conclusions:**

This study demonstrated that extended working hours increased the risk of mental disorders among nurses during the coronavirus disease pandemic, particularly in those who worked more than 60 h per week. These findings enrich the literature on mental disorders and demonstrate a critical need for additional studies investigating intervention strategies.

## Background

Anxiety is a common and disabling condition characterized by feeling of nervousness and fear, including generalized anxiety, panic, and social anxiety disorders [1, 2]. According to the Global Burden of Disease databases, anxiety disorder was estimated as prevalent among approximately 4% of the population worldwide in 2017 [3]. Depression is also a common mental disorder typically accompanied by depressed mood, decreased interest or loss of pleasure, and self-blame [2, 4]. It is estimated that 3.8% of the general population suffers from depression worldwide [5]. However, in a systematic study including 22 provinces in China with 52,592 participants, depressive symptoms among nurses were estimated at 43.83% [6]. This was significantly higher than those of other countries [7]. Poor mental health often affects regular activities and probably results in poor professional performance and suicide [8–10]. Moreover, it could also cause abnormal physical disorders, such as inflammation [1, 9].

An eight-hour workday legally began in 1868 in the United States [11], and exceeding 40 h per week was defined as long working hours [12]. Long working hours remain an essential issue for employees. In recent years, many countries restricted working hours. For example, the European Working Time Directive (EWTD) implemented a policy in 1998 with a maximum workweek of 48 h. Denmark, Sweden, and Germany have been compliant with the EWTD for several years [13]. Long working hours usually result in physical illness and mental disorders [14–17].

After a five-year follow-up, 2,960 full-time employees from the prospective Whitehall II cohort study of British civil servants were recruited. The results showed that weekly working hours > 55 h predicted subsequent depression and anxiety; each 10-hour increase was associated with a 17% and 22% increase in the risk of depression and anxiety, respectively [14]. Another cross-sectional research conducted in Australia, including 12,252 participants, reported that the at-risk participants (junior doctors) who worked > 55 h per week were twice as likely to develop depression and anxiety than those who worked 40–44 h [15]. However, studies on nurses are scarce. In 2009, ten years before the COVID-19 pandemic, a multi-center cross-sectional study included 3,474 nurses in public hospitals of southern China was conducted. Gong, Yanhong et al. [18] found that an estimated 38% of nurses had depressive symptoms measured using the 20-item SDS, and depressive symptoms were associated with frequent workplace violence, long working hours (more than 45 h per week), frequent night shifts (two or more per week), and specific departments (surgical and pediatric department). In another pre-COVID-19 pandemic period study conducted in 2016, and included 291 nurses from three general hospitals in Korea [19], they found that the emotional labor of nurses with long working hours influenced depression measured using CES-D. The studies addressing this issue are limited, and the conclusions conflict. Notably, there was no study conducted during the COVID-19 pandemic period.

Nurses were likely to experience mental disorders due to high levels of occupational stress [20, 21]. They inevitably worked long hours, had time constraints, and irregular schedules [7, 22]. Mental disorders are a critical issue for nurses and the safety of patients [9]. During the COVID-19 pandemic, individuals were restricted to their homes to prevent the spread of the virus. Social communication was limited, contributing to the prevalent incidence of mental illness disorders among healthcare workers [23]. Therefore, based on a large cross-sectional study, we aim to investigate the association between long working hours and mental disorders in Chinese nurses during the COVID-19 pandemic.

## Methods

### Study design

**Fig. 1:**
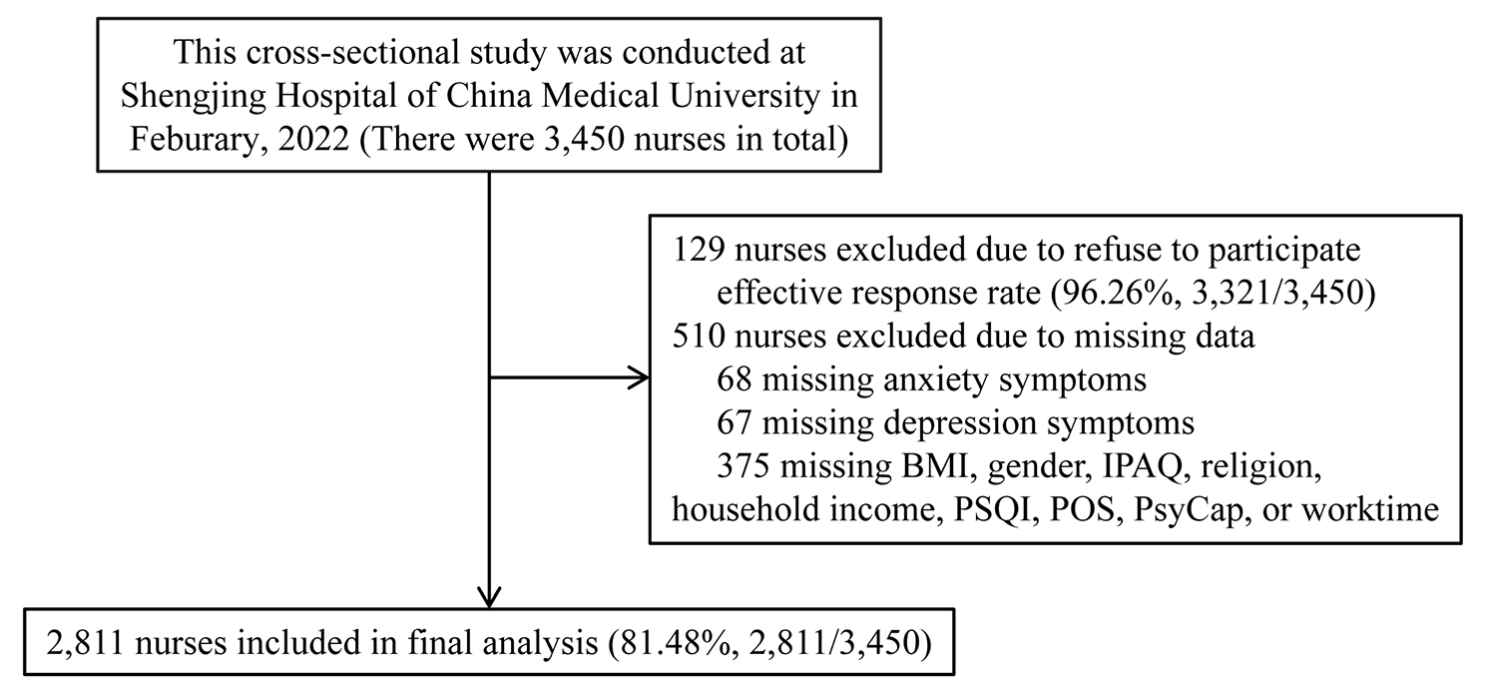
Flowchart of the study

This cross-sectional study was conducted at Shengjing Hospital of China Medical University from March 2022 to April 2022. Shengjing Hospital is a tertiary hospital and is the third-largest hospital in China, with more than 6,700 beds. Three thousand four hundred fifty nurses (all nurses working in this hospitals) were enrolled in the present study. Finally, effective responses were obtained from 2,811 individuals (effective response rate: 81.48%). A set of self-administered questionnaires was adopted. Participants completed a structured questionnaire within 20 to 25 min. A flow chart illustrating the process is detailed in Fig. 1.

### Inclusion and exclusion criteria

The inclusion requirements were as follows: occupationally active (actually working) nurses who were employed in hospitals. The following exclusion criteria were applied: nurses who had worked for less than three months; the disease history of any mental disease, especially anxiety, and depression.

### Measurement of characteristics

Demographic factors included age, gender, and body mass index (BMI, kg/m2) in this investigation. Dietary habits included alcohol consumption, and coffee consumption; life-related factors included smoking status, sleep quality (PSQI, Pittsburgh sleep quality index scores), physical activity (IPAQ, International Physical Activity Questionnaire, Mets hour/week), religion, marital status, number of siblings, monthly household income (RMB, yuan), experienced major life events, history of chronic disease, and frequent friend visits.

Work-related characteristics include job duration, specialization, and night shifts. The smoking behavior was classified as a current smoker ( 1 cigarette per day for six months), past smoker (quit six months ago), or never smoker. The categories for alcohol consumption and coffee consumption include current drinker ( 1 time per day and within the last six months), former drinker (stopped drinking within the last six months), and never drinker. Significant life events experienced included separation/divorce, death or serious illness of close family members, serious injury/traffic accident, violence (including workplace violence, such as insults, threats, and physical attacks), unemployment, natural disasters, death or serious illness of a partner, serious conflict with family, medical disputes, and income decrease/debt. Evaluation of physical illness history was based on responses (yes or no) to questions regarding a disease history (including Cataract, purpura, osteoarthritis, osteoporosis, endometrial polyps, hysteromyoma, breast nodules, gallbladder polyps, fatty liver, arrhythmia, irregular menstruation, asthma, lumbar disc protrusion, gastric ulcer, breast hyperplasia, Hashimoto thyroiditis, ankylosing spondylitis, thyroid tumor, hyperthyroidism, and hypothyroidism). The term “exposure to the COVID-19 pandemic” refers to nurses who may come into touch with COVID-19-suspected or -positive patients, or who may encounter a circumstance requiring COVID-19 quarantine.

Physical activity (PA) in the most recent week was assessed using the short form of the International Physical Activity Questionnaire [24], The Cronbach’s α coefficient was 0.922. Sleep quality was measured by the Pittsburgh sleep quality index [25], The Cronbach’s α coefficient was 0.901. The Chinese version of the Perceived Organization Support Questionnaire (POS) was utilized to measure the level of organization support [26]. The Cronbach’s α coefficient for POS was 0.9261. PsyCap was evaluated by the Chinese version of the 24-item Psychological Capital Questionnaire (PCQ) [27, 28]. The Cronbach’s α coefficients for self-efficacy, hope, resilience, and optimism were 0.921, 0.936, 0.920, and 0.900. Depressive symptoms were measured by clinically validated scales for Patient Health Questionnaire (PHQ-9) [29]. The presence of major depression was defined as a PHQ-9 score ≥ 10. Anxiety was measured by the General Anxiety Disorder (GAD-7) questionnaire (cutoff value ≥ 10 was defined as anxiety) [30]. The Cronbach’s α coefficients for PHQ-9 and GAD-7 were 0.951 and 0.928, respectively.

### Statistical analysis

Windows SPSS 22.0 was used to analyze the data (SPSS Inc., Chicago, IL, USA). Median values with interquartile range for continuous variables were given. As a count, categorical variables were reported as numbers (percentages). Independent samples Student’s t-test was utilized to compare the means of two continuously distributed normally distributed variables. The Mann-Whitney U test was used to compare the means of two continuous, non-normally distributed variables. For categorical variables, the χ^2^ test or Fisher’s exact test was employed.

Based on the distribution for all participants, worktime weekly was divided into four quartiles (≤ 40 h, 41–60 h, 61–80 h, and ≥ 81 h ) for further analysis. Using binary unconditional logistic regression analysis, the relationships between quartile categories of worktime weekly and mental disorders status (depression and anxiety) were investigated. The presence of mental disorders served as the dependent variable, whereas worktime weekly served as the independent variable.

The crude model was utilized to obtain the crude odds ratios (OR) without adjusting any variable, and model 1 was further adjusted for age, gender, and BMI. Model 2 additionally adjusted for baseline variables that were considered clinically relevant or that had a P value < 0.10 in the univariate analysis, including BMI, alcohol habit, sleep quality, having siblings, experienced major events, visiting friends constantly, years of employment, working duration, perceived organization support, psycap-efficacy, psycap-hope, psycap-resiliency and psycap-optimism for depression, and adjusted sage, BMI, sleep quality, physical activity, marital status, have siblings, experienced major events, history of chronic disease, visiting friend constantly, years of employment, speciality, working duration, perceived organization support, psycap-efficacy, psycap-hope, psycap-resiliency and psycap-optimism for anxiety. Model 3 adjusted for all baseline variables.

Adjusted odds ratios and 95% confidence intervals (CI) were calculated using binary unconditional logistic regression after controlling for variables. Using the median value of each quartile as a continuous variable, a linear trend across increasing quartiles was examined. All P values were two-tailed, and when P < 0.05, the difference was statistically significant.

## Results

There were 2,811 nurses finally included in this study, with a median age of 35 years and a median BMI of 21.83 kg/m^2^, and 94.20% of participants were women. Moreover, the percentage of working hours weekly less than 40 h, 41–60 h, 61–80 h, and more than 80 h were 35.5% (998), 61.2% (1,720), 0.6% (16), and 2.7% (77), respectively. The incidence of nurses who experienced depression and anxiety was 7.80% (219/2,811) and 6.70% (189/2,811), respectively.

In the univariate analysis of depression, participants with higher BMI, poor sleep quality, and lower scores of perceived organization support, efficacy, hope, resiliency, and optimism were more likely to suffer from depression; A higher incidence of participants with depression had current alcohol habits, had siblings, experienced major events, visited with friends rarely, worked for more than five years, and worked for more than 40 h weekly. In the univariate analysis of anxiety, participants with older age, higher BMI, poor sleep quality, lower weekly physical activity, lower scores of perceived organization support, efficacy, hope, resiliency, and optimism were more likely to suffer from anxiety. A higher rate of participants with anxiety got married / cohabitation, had siblings, experienced major events, had a history of chronic illness, visited friends rarely, participated in a job for more than five years, worked for more than 40 h weekly, and was employed in the surgical department. All the variables mentioned above were the statistical difference in univariate analysis; see detailed in Table 1.

**Table 1:** Baseline characteristics according to mental health in the cohort analysis (n = 2,811)

Variables	Total	Depression			Anxiety		
		Depression	Without depression	p	Anxiety	Without anxiety	p
**Number** (%)	2,811	219 (7.80)	2,592 (92.20)		189 (6.70)	2,622 (93.30)	
**Demographic characteristics**							
Age (years)	35.00 (32.00,37.00)	35.00 (32.00, 37.00)	35.00 (32.00, 37.00)	0.810	35.00 (33.00, 38.00)	35.00 (32.00, 37.00)	< 0.001
Gender (female)	2,649 (94.20)	210 (95.90)	2,439 (94.10)	0.277	180 (95.20)	2,469 (94.20)	0.542
BMI (kg/m^2^)	21.83 (20.07,23.88)	22.57 (20.76, 24.38)	21.76 (19.96, 23.80)	0.002	22.66 (20.76, 24.09)	21.77 (19.95, 23.83)	0.005
**Dietary habits**							
Smoking habit				0.659			0.431
Current	36 (1.30)	3 (1.40)	33 (1.30)		3 (1.60)	33 (1.30)	
Former	24 (0.90)	3 (1.40)	21 (0.80)		3 (1.60)	21 (0.80)	
Never	2,751 (97.90)	231 (97.30)	2,538 (97.90)		183 (96.80)	2,568 (97.90)	
Alcohol habit				0.039			0.624
Current	192 (6.80)	24 (11.00)	168 (6.50)		15 (7.90)	177 (6.80)	
Former	144 (5.10)	12 (5.10)	144 (5.40)		9 (4.80)	135 ( 5.10)	
Never	2,475 (88.00)	198 (83.50)	2,355 (88.00)		165 (88.10)	2,310 (88.10)	
Coffee habit				0.606			0.621
Current	768 (27.30)	60 (27.40)	708 (27.30)		45 (23.80)	723 (27.60)	
Former	423 (15.00)	39 (17.80)	384 (14.80)		36 (19.00)	387 (14.80)	
Never	1,620 (57.60)	120 (54.80)	1,500 (57.90)		108 (57.10)	1,512 (57.70)	
**Life related factors**							
Sleep quality (PSQI scores)	5.00 (3.00,8.00)	10.00 (8.00, 12.00)	5.00 (3.00, 7.00)	< 0.001	10.00 (7.00, 12.00)	5.00 (3.00, 7.00)	< 0.001
Physical activity (IPAQ Mets×hour/week)	18.60 (3.65,52.80)	14.60 (0.00, 46.20)	19.30 (4.00, 53.77)	0.824	20.50 (0.00, 87.10)	18.39 (4.00, 51.30)	0.006
Have religions (yes)	93 (3.30)	90 (3.50)	3 (1.40)	0.107	3 (1.60)	90 (3.40)	0.182
Marital status				0.117			0.099
Single	543 (19.30)	48 (21.90)	495 (19.10)		30 (15.90)	513 (19.60)	
Married/cohabitation	2220 (79.00)	171 (78.10)	2049 (79.10)		153 (81.00)	2,067 (78.80)	
divorce/separation/widow	48 (1.70)	0 (0.00)	48 (1.90)		6 (3.20)	42 (1.60)	
Have siblings (yes)	2,013 (71.60)	168 (76.70)	1,845 (71.20)	0.082	150 (79.40)	1,863 (71.10)	0.015
Household income (Yuan/month)				0.905			0.258
< 5,000	15 (0.50)	3 (1.40)	12 (0.50)		0 (0.00)	15 (0.60)	
≧ 5,000, < 10,000	462 (16.40)	33 (15.10)	429 (16.60)		39 (20.60)	423 (16.10)	
≧ 10,000	2,334 (83.0)	183 (83.60)	2,151 (83.00)		150 (79.40)	2,184 (83.30)	
Experienced major events (yes)	1,443 (51.30)	147 (67.10)	1,296 (50.00)	< 0.001	117 (61.90)	1,326 (50.60)	0.003
History of chronic disease (yes)	522 (18.60)	42 (19.20)	480 (18.50)	0.810	45 (23.80)	477 (18.20)	0.056
Visiting friend constantly (no)	75 (2.70)	12 (5.50)	63 (2.40)	< 0.001			< 0.001
**Work related factors**							
Years of employment				0.100			0.067
< 5 years	384 (13.70)	18 (8.20)	366 (14.10)		12 (6.30)	372 (14.20)	
5–10 years	1,137 (40.40)	96 (43.80)	1,041 (40.20)		87 (46.00)	1,050 (40.00)	
> 10 years	1,290 (45.90)	105 (47.90)	1,185 (45.70)		90 (47.60)	1,200 (45.80)	
Speciality				0.270			0.004
Surgery	1,209 (43.00)	105 (47.90)	1,104 (42.60)		120 (63.50)	1,089 (41.50)	
Internal medicine and others	321 (11.40)	24 (11.00)	297 (11.50)		18 (9.50)	303 (11.60)	
Obstetrics and Gynecology	342 (12.20)	15 (6.80)	327 (12.60)		6 (3.20)	336 (12.80)	
Pediatrics	255 (9.10)	6 (2.70)	249 (9.60)		3 (1.60)	252 (9.60)	
Others	684 (24.30)	69 (31.50)	615 (23.70)		42 (22.20)	642 (24.50)	
Worktime duration (hours/week)				< 0.001			< 0.001
< 40 h	998 (35.50)	64 (29.20)	934 (36.00)		55 (29.10)	943 (36.00)	
41–60 h	1,720 (61.20)	134(61.20)	1,586(61.20)		113(59.80)	1,607(61.30)	
61–80 h	16 (0.60)	6 (2.70)	10 (0.40)		5 (2.60)	11 (0.40)	
> 80 h	77 (2.70)	15 (6.80)	62 (2.40)		16 (8.50)	61 (2.30)	
Night shifts (more than 3 times/month)	1,590 (56.60)	135 (61.60)	1,455 (56.10)	0.115	117 (61.90)	1,473 (56.20)	0.126
Exposure to the COVID-19 (yes)	315 (11.20)	30 (13.60)	285 (11.00)	0.135	24 (12.70)	291 (11.10)	0.160
**Psychological characteristics**							
POS scores	51.00 (44.00,57.00)	44.00 (36.00, 50.00)	51.00 (45.00, 57.00)	< 0.001	46.00 (40.00, 51.00)	51.00 (45.00, 57.00)	< 0.001
PsyCap-efficacy (scores)	29.00 (24.00,31.00)	24.00 (21.00,26.00)	30.00 (24.14, 32.75)	< 0.001	24.00 (21.00, 28.00)	30.00 (24.00, 32.00)	< 0.001
PsyCap-hope (scores)	30.00 (24.00,32.00)	23.69 (21.00, 26.00)	30.00 (25.00, 32.00)	< 0.001	24.00 (20.00, 29.00)	30.00 (24.00, 32.00)	< 0.001
PsyCap-resiliency (scores)	27.00 (24.00,31.00)	24.00 (21.51, 27.00)	27.00 (24.00, 31.00)	< 0.001	25.00 (23.00, 27.00)	27.00 (24.00, 31.00)	< 0.001
PsyCap-optimism (scores)	26.00 (23.00,28.00)	23.00 (22.00, 25.00)	26.00 (24.00, 29.00)	< 0.001	23.00 (22.00, 24.00)	26.00 (24.00, 29.00)	< 0.001

Categorical variables were reported as the number (percentage). Independent samples Student’s t-test was used to compare the mean of two continuous normally distributed variables, and the Mann-Whitney U test was used to compare the mean of two continuous non-normally distributed variables, The χ2 test or Fisher’s exact test was used for categorical variables

Abbreviations: BMI, body mass index; PSQI, Pittsburgh sleep quality index, IPAQ, International Physical Activity Questionnaire; COVID-19, Coronavirus Disease 2019; POS, Perceived Organization Support; PsyCap, Psychological Capital

To explore the relationship between the working hours weekly and depression, we categorized the working hours weekly into four levels; after multivariate analysis (Model 2), compared with the lowest reference, the ORs and 95% CI across quartiles were 0.98 (0.69, 1.40), 10.58 (2.78, 40.32) and 1.79 (0.81, 3.97), respectively, and the *P* for trend was 0.002. For anxiety, after multivariate analysis (Model 2), the ORs across quartiles were 0.87 (0.59, 1.30), 8.69 (2.13, 35.46), and 2.67 (1.26, 5.62), respectively, and the *P* for trend was 0.008. see detailed in Table 2.

**Table 2:** Association between quartiles of worktime weekly and mental health in the cohort analysis (n = 2,811)

	Quartiles of worktime duration weekly (range, n = 2811)				*P* for trend ^a^
Worktime weekly (hours)	Level 1 (≤ 40)	Level 2 (41–60)	Level 3 (61–80)	Level 4 (≥ 81)	
**Depression**					
No. of participants	998	1720	16	77	
No. of depression	64	134	6	15	
Crude model	Reference	1.23 (0.91, 1.68) ^b^	8.76 (3.09, 24.86)	3.53 (1.90, 6.55)	< 0.001
Adjusted model 1 ^c^	Reference	1.23 (0.90, 1.68)	9.84 (3.45, 28.11)	1.07 (1.03, 1.11)	< 0.001
Adjusted model 2 ^d^	Reference	0.98 (0.69, 1.40)	10.58 (2.78, 40.32)	1.79 (0.81, 3.97)	0.002
Adjusted model 3 ^e^	Reference	0.85 (0.59, 1.24)	9.30 (2.33, 37.17)	1.57 (0.70, 3.54)	0.003
**Anxiety**					
No. of participants	998	1720	16	77	
No. of anxiety	55	113	5	16	
Crude model	Reference	1.21 (0.87, 1.68) ^b^	7.79 (2.62, 23.22)	4.50 (2.43, 8.31)	< 0.001
Adjusted model 1 ^c^	Reference	1.27 (0.91, 1.78)	10.52 (3.48, 31.82)	4.73 (2.54, 8.79)	< 0.001
Adjusted model 2 ^d^	Reference	0.87 (0.59, 1.30)	8.69 (2.13, 35.46)	2.67 (1.26, 5.62)	0.008
Adjusted model 3 ^e^	Reference	0.92 (0.62, 1.37)	9.61 (2.34, 39.51)	3.03 (1.42, 6.48)	0.003

^a^ Multiple Logistic regression analysis

^b^ Odd ratio (95% confidence interval) (all such values)

^c^ Adjusted for age, sex, and body mass index

^d^ Additionally adjusted alcohol habit status, sleep quality status, have siblings, experienced major events, visiting friend constantly rarely, years of employment, specially, years of employment, working duration, perceived organization support, psycap-efficacy, psycap-hope, psycap-resiliency and psycap-optimism for depression based on Model 1. And adjusted sleep quality status, physical activity status, marital status, have siblings, experienced major events, history of chronic disease, visiting friend constantly rarely, years of employment, working duration, perceived organization support, psycap-efficacy, psycap-hope, psycap-resiliency and psycap-optimism for anxiety based on Model 1

e Additionally adjusted for all baseline variables

## Discussion

Long working hours is a common risk factor of mental disorders, however, studies on nurses are scarce, particularly during the COVID-19 pandemic. Therefore, we aimed to investigate the association between long working hours and mental disorders in nurses based on a large cross-sectional study in China during the COVID-19 pandemic. We found that there was a positive relationship between long working hours and mental disorders, particular in nurses who worked more than 60 h per week.

Similarly, a prospective cohort study of British civil servants, which included 2,960 full-time employees, demonstrated that long working hours weekly was a risk factor for the development of depressive and anxiety symptoms. Furthermore, the risk of anxiety and depression was much higher when the work hours per week > 55 h for women [14]. A multi-center cross-sectional study included 3,474 nurses in public hospitals in southern China. Gong, Yanhong et al. [18] found that depressive symptoms were associated with long working hours (more than 45 h per week). Excessive working hours have a significantly negative impact on mental health, particularly in medical workers. A cross-sectional study in Australia with 12,252 physicians, using a random sample in 2013, also confirmed a strong positive association between working > 50 h per week and poor mental health outcomes among junior doctors [15].

On the contrary, a meta-analysis aimed to examine whether excessive working hours were associated with depressive disorder among workers, and it demonstrated that overtime work was associated with a minor, non-significant, elevated risk of depressive disorder (*P* = 0.575). The association tended to be greater for women [31]. The difference in professions could explain this discrepancy. Another meta-analysis included approximately 190,000 participants from 28 prospective cohort studies in 35 countries. It also indicated only a moderate association between long working hours and the onset of depressive symptoms in Asia and was minor in Europe [32]. These results could be attributed to cultural and occupational health policy differences between Asia and Europe. A multi-center, cross-sectional study including 1,343 medical residents from eight hospitals in Northeast China was conducted from December 2019 to February 2020, Bai S et al., found that experienced long work hours was associated with anxiety in univariate analysis (P<0.001), but is not statistically significant after multivariate analysis (P = 0.614) [33]. The discrepancy could be caused by different occupational participants.

The plausible mechanism is that excessive working hours can usually cause sleep deprivation, resulting in an increase in inflammatory indicators in the body. A study that used 13 healthy young men as the experimental group with restricted sleep duration and six as the control during a five-day experimental period showed that both IL-6 and C-reactive protein (CRP) significantly increased in the experimental group members [34]. Moreover, shift work can disturb sleeping patterns, thereby leading to insulin resistance and the immune system disturbance, resulting in inflammation [35, 36], which plays an essential role in developing depression [37, 38].

## Limitation

There are several limitations in this study. First, it was a cross-sectional study, therefore, we were unable to assess the causal relationships among the variables. A longitudinal study should be conducted to verify our conclusions. Second, we did not perform subgroup analysis by gender due to the limited number of male nurses (5.76%). Third, we collected data using self-report questionnaires, which might lead to recall or reporting bias. Fourth, we identified mental health issues using the PHQ-9 and GAD-7 questionnaires instead of clinical diagnoses. Fifth, the participants were recruited in only one hospital of china. Thus, our findings cannot be generalized nationally and to other countries. Therefore, a national study should be conducted in the future. Finally, although we found that extended working hours increased the risk of mental disorders among nurses during the coronavirus disease pandemic, particularly in those who worked more than 60 h per week, the number of participants in this stage (≥ 60 h) is not large enough. Therefore, it is difficult to draw a conclusion from this study. However, this is the first study on the association between long working hours and mental health in nurses during the COVID-19 pandemic. Furthermore, this large cross-sectional study adjusted for comprehensive confounding factors.

## Conclusions

This cross-sectional study demonstrated that extended working hours increased the risk of mental disorders among nurses during the COVID-19 pandemic, particularly in those who worked more than 60 h per week. These findings enrich the existing literature on mental disorders and demonstrate a critical need for additional studies investigating intervention strategies.

## Data Availability

The datasets used in the current study are available from the corresponding author on reasonable request.
